# Assessing circum-maxillary sutures maturation by CBCT: a diagnostic indicator for skeletal age and treatment timing for class III malocclusion

**DOI:** 10.3389/froh.2026.1749578

**Published:** 2026-01-30

**Authors:** Zhili Dong, Yue Zeng, Xiaoxing Kou, Liping Wu, Hong Hong

**Affiliations:** 1 Hospital of Stomatology, Guangdong Provincial Clinical Research Center of Oral Diseases, Sun Yat-sen University, Guangzhou, China; 2 Department of Stomatology, The People’s Hospital of Guangxi Zhuang Autonomous Region & Guangxi Academy of Medical Sciences, Nanning, China; 3 South China Center of Craniofacial Stem Cell Research, Guanghua School of Stomatology, Sun Yat-Sen University, Guangzhou, China

**Keywords:** cervical vertebrae maturation stages, maxillary expansion and protraction, midpalatal suture, skeletal class III malocclusion, zygomaticomaxillary suture

## Abstract

**Objectives:**

Evaluation of skeletal age is essential in determining the timing of maxillary expansion and protraction for adolescent skeletal Class III patients. The aim of this study is to investigate the correlation among the maturation stage of cervical vertebrae (CVMS), midpalatal suture (MPSS) and zygomaticomaxillary suture (ZMSS) and evaluate the possibility of predicting the outcome of maxillary expansion and protraction using circum-maxillary suture maturation stage.

**Methods:**

Cone beam computed tomography (CBCT) scans and lateral cephalography images of 260 adolescents were analyzed to determine the correlation among different maxillary sutures maturation stages and CVMS. Statistical analysis included the Mann–Whitney, Wilcoxon, and Fisher's exact tests. The level of significance was 5% (*P* = 0.05).

**Results:**

The distribution difference of MPSS and ZMSS is statistically significant between different age groups (*P* < 0.05), and two sutures maturation stages both showed a trend of increasing maturation with age. CVMS, MPSS and ZMSS were all significantly correlated (*P* < 0.001). Positive likelihood ratio analysis revealed strong diagnostic associations (LHR >10) between specific CVMS stages and corresponding suture maturation levels, particularly for the early and late maturation stages.

**Conclusion:**

This study verified the efficacy of using the maturational stages of maxillary sutures to assess growth and development. MPSS shows a strong correlation with ZMSS and can serve as a useful reference for estimating ZMSS maturation, supporting a combined assessment approach. The maxillary suture maturational stages can serve as an index to evaluate the intervening time and outcomes of maxillary protraction with palatal expansion in juveniles.

## 1 Introduction

Class III malocclusion affects approximately 5% to 15% of our population, with skeletal Class III malocclusion being recognized as one of the most challenging problems for orthodontic therapy, often necessitating early intervention (1). Clinically, Class III malocclusion associated with maxillary deficiency manifests as a retrusion of the nasomaxillary area and dentoalveolar abnormalities in the transverse dimension of the maxilla. McNamara et al. suggested that over sixty percent of patients with Class III malocclusion exhibit maxillary retrognathism (2). The main purpose of early intervention is to remove the impediments for craniofacial bone growth and to improve the occlusal relationship, which results in better orofacial function and facial esthetics (3, 4). Maxillary protraction combined with transverse palatal expansion (MP/TPE) is widely acknowledged as a promising orthopedic approach for addressing early skeletal Class III cases with maxillary deficiency (5).

However, the optimal timing of initiating MP/TPE remains controversial. Different chronological ages like 8 years, 9 years and 10 years of age have been proposed, though some authors considered choosing timing by chronological age was unreliable (6, 7). Due to the lack of a reliable parameter to evaluate the maturation stage of the sutures, many professionals tend to choose miniscrew-assisted rapid palatal expansion and surgically assisted maxillary expansion in class III patients with maxillary deficiency despite their higher cost and potential side effects such as ulcerations, erythema, itching, and discomfort in palatal mucosa (8, 9). As a non-surgical orthopedic treatment, MP/TPE exerts direct and continuous orthopedic forces on maxilla and transmitted to the circum-maxillary sutures, resulting in the separation of circum-maxillary sutures and bone remodeling. However, as patients age, the circum-maxillary sutures gradually close and more integration can impede the reconstruction of sutures, causing undesirable side effects and even failure of MP/TPE (10, 11). Therefore, to achieve best skeletal effects in minors with maxillary deficiency, accurately predicting the optimal timing of MP/TPE treatment is indispensable and widely debated among orthodontists.

The CVM method was initially proposed by Lamparski as a growth indicator of individual skeletal maturity, which was further refined by Baccetti, Franchi and McNamara for the purposes of orthodontic diagnosis and treatment (12). The reliability and validity of CVMS in evaluating the pubertal peak and growing stage of skeletal growth had been demonstrated by extensive researches (13, 14). In orthodontics, CVMS are commonly adopted in predicting the maturation stage of circum-maxillary sutures, but data from several researchers suggested that CVMS is more consistent with the growth of mandible rather than maxilla (15, 16). In addition, Perinetti et al. have shown that relying solely on the CVM method for treatment timing decisions is not entirely reliable and recommended its combined use with other procedures (17). Beit et al*.* analyzed 730 sets of radiographs (cephalogram and hand-wrist) to examine the correlation between cervical vertebral morphology and skeletal age. They concluded that cervical spine evaluation offers no advantage over chronologic age in either assessing skeletal age or predicting the pubertal growth spurt (18). Therefore, exploring new approaches to assess the growth stage of maxilla is essential for evaluating the optimum timing for maxillary expansion therapy.

Previous researches have shown that success of maxillary protraction and extension is strongly related to the maturation of circum-maxillary sutures. Among these sutures, the orientations of ZMSS and MPSS closely align with the directions of the applied force system used in MP/TPE procedures. Angelieri et al. found that ZMSS and MPSS show an increasing interdigitation and complexity from the infantile through the adolescent stages (19). In the past, the maturation of the circum-maxillary sutures is considered to be solely influenced by the patient's chronological age. But recent studies suggested that great variability exists among individuals due to factors such as the population's ethnicity, nutritional conditions, endocrine function and surrounding anatomic structures (20, 21). While the methodologies for assessing MPS and ZMS maturation are established, the applicability of existing data across diverse ethnic groups is limited. A recent systematic review by Gonzalvez Moreno et al. underscored the high variability in suture maturation across different populations, concluding that ethnicity-specific data are essential as findings from one population may not be directly transferable to another (22). Currently, comprehensive data on circum-maxillary suture maturation in a large Chinese adolescent population is scarce. Therefore, the primary aim of this study is to investigate the correlation among CVMS, MPS, and ZMS maturation stages within a Chinese cohort. This will provide a population-specific diagnostic indicator and evaluate the potential of using MPS maturation to predict ZMS status, thereby clarifying the optimal timing for maxillary expansion and protraction therapy in this demographic.

## 2 Materials and methods

### 2.1 Ethics statement

All of the subjects were diagnosed at the orthodontic department of the Hospital of Stomatology, Sun Yat-sen University. This study was performed in accordance with the Declaration of Helsinki and approved by the Medical Ethics Committee of the Hospital of Stomatology, Sun Yat-sen University, (Protocol Title: The Significance of Maturational Stages Of Circum-maxillary Sutures In Assessing The Outcomes Of Maxillary Expansion And Protraction Of Juveniles In Guangdong, No. KQEC-2021-43-02). The Ethics Committee of the Hospital of Stomatology of Sun Yat-sen University waived the need for individual informed consent, as this study had a non-interventional retrospective design, and all the data used in this study were anonymized before analysis.

### 2.2 Participants in the retrospective study and CBCT analysis

This retrospective cross-sectional study included 260 adolescents (130 males, 130 females; aged 6–18 years). CBCT scans and lateral cephalograms were acquired for all participants. All CBCT scans were obtained previously as part of standard diagnostic care, following strict clinical justification protocols. The primary indications for CBCT imaging included the comprehensive assessment of impacted teeth (e.g., canines, third molars), root positions, airway space and temporomandibular joint morphology. No scan was performed solely for research purposes, adhering to the ALARA (As Low As Reasonably Achievable) principle for radiation safety. The inclusion criteria were as follows: (1) age from 6 to 18 years; (2) no systematic disease; (3) no craniofacial trauma appeared. Subjects with craniofacial malformations (including cleft lip or palate), lesions in the region of the suture, history of surgical procedures and previous orthodontic treatment were excluded from the study.

The suture stages of the ZMS and MPS were analyzed. The CBCT image processing was performed using Carestream Dental CS 3D Imaging software (Carestream Health, Rochester, NY, USA). The suture stage of the ZMS and MPS were analyzed. The adjustment of the patient's head in the 3 planes of space and the selection of the slice for evaluation the sutures maturations were performed according to the protocol described by Angelieri et al. (19, 23).

The maturation stages for each suture were defined as follows:

For the midpalatal suture, Stage A is defined as a straight high-density suture line with no or little interdigitation; Stage B shows a scalloped high-density suture line, which may include areas with two parallel lines; Stage C appears as two parallel, scalloped, high-density lines close to each other and separated by small low-density spaces; Stage D involves fusion in the palatine bone, where the suture is not visible; and Stage E signifies fusion in the maxillary bone, with no evidence of the suture (24).

**Figure 1:**
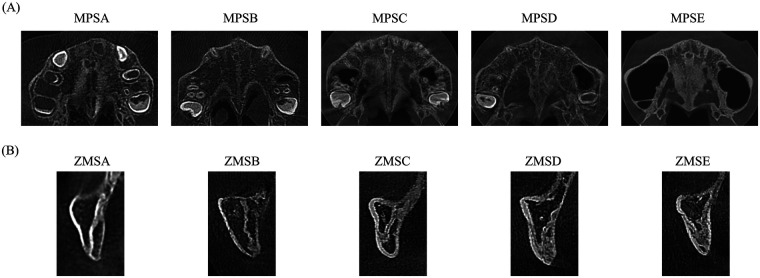
Representative CBCT images illustrating the five maturation stages **(A–E)** of the **(A)** midpalatal suture (MPS) and **(B)** zygomaticomaxillary suture (ZMS).

For the zygomaticomaxillary suture, Stage A represents a uniform high-density suture line with no or little interdigitation; Stage B exhibits a scalloped appearance of the high-density suture line; Stage C is characterized by two parallel, scalloped, high-density lines separated in some areas by small low-density spaces; Stage D indicates fusion in the inferior portion of the suture; and Stage E denotes complete fusion (19). Figure 1 presents the representative CBCT images depicting the five maturation stages (A–E) for both the MPS (A) and the ZMS (B).

The CVMs were analyzed according to the protocol described previously by Baccetti et al. using lateral cephalograms used in orthodontic diagnosis and treatment planning (25). Lateral cephalograms were acquired separately using the same digital cephalometric system x-ray machine (KODAK 8000C, Carestream, Canada) under same settings (tube voltage: 80 kV, tube current: 10 mA). The radiographs were captured with patients in the natural head position, teeth in centric occlusion, and lips relaxed.

To reduce observer bias, the two examiners who performed the suture maturation assessments were blinded to the patients' age and sex. All CBCT scans were anonymized and randomly coded by a third investigator prior to evaluation. The examiners assessed only the coded images without access to demographic or clinical data.

### 2.3 Statistical analysis

Group comparisons for the distribution of MPSS and ZMSS across age groups were performed using Chi-square or Fisher's exact test, as appropriate. *Post hoc* pairwise comparisons were conducted when overall significance was detected. Pearson and Spearman correlation analysis were performed to investigate the correlations among the CVMS, ZMSS and MPSS. The relationship among CVMS, ZMSS and MPSS was evaluated with the positive likelihood ratio (LHR), a measure of diagnostic performance. A positive LHR of 10 or more for any CVM stage was considered a reliable indicator for the diagnosis of any of the maturational stages of the midpalatal suture. A *P* value less than 0.05 was considered statistically significant. All tests were conducted using SPSS software (IBM SPSS version 19.0, Armonk, NY, USA).

The sample size was calculated using G*Power 3.1.9.7 (Heinrich-Heine-University, Düsseldorf, Germany). Based on a previous correlation study between cervical vertebral maturation and midpalatal suture maturation, a minimum expected correlation coefficient of *r* = 0.4 was adopted. With an alpha level of 0.05 and a statistical power of 80%, the analysis indicated that at least 46 subjects per group were required (26).

Assessment of intra and inter-examiner reproducibility was performed 30 days after the initial measurements. Two investigators performed the analysis by randomly selecting 30 CBCTs coded by a third investigator; CVMS, MPSS and ZMSS were all evaluated. The assessment of both intra and inter-examiner reproducibility had Kappa coefficient values higher than 0.80.

## 3 Results

### 3.1 Midpalatal suture maturation stage in adolescent subjects

**Figure 2:**
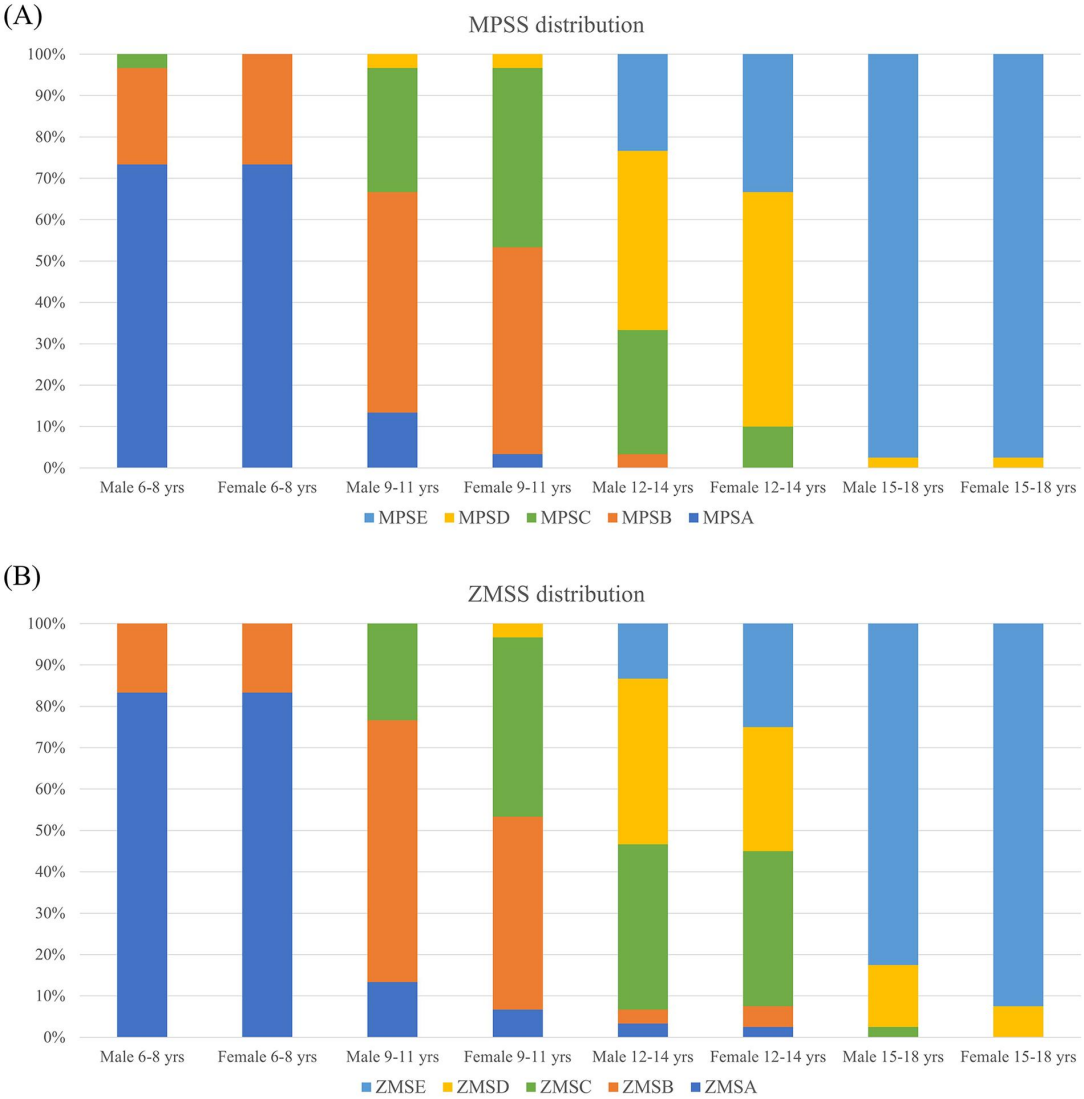
Distribution of maturation stages for the **(A)** midpalatal suture (MPSS) and **(B)** zygomaticomaxillary suture (ZMSS) across different age groups and genders. Stacked bar charts show the relative proportion of each stage (A to E) within the specified demographic subgroups.

As age increases, MPSS shows a gradually developing trend from stage A to stage E in male and female adolescents aged 6–18 (Figure 2A). Stage A was the most prevalent MPSS of males and females at age 6–8; stage A, B, C, and D of MPSS were observed in both gender at 9–11, but in females, there was a higher proportion of MPSS in stages B and C. In males at 12–14, MPSS are mainly distributed in stages C, D, and E; while those of females mainly distributed in stages D and E. Almost all MPSS of males and females aged 15–18 are stage E, and there is a statistically significant difference in the distribution of MPSS between different age groups (Table 1).

**Table 1:** The distribution of MPSS at different ages in all subjects.

Gender	Age	MPSS					Total
		A	B	C	D	E	
Male	6**–**8^a^^,^^b^^,^^c^	22 (73.33%)	7 (23.33%)	1 (3.33%)	0	0	30
	9**–**11^a^^,^^d^^,^^e^	4 (13.33%)	16 (53.33%)	9 (30.00%)	1 (3.33%)	0	30
	12**–**14^b^^,^^d^^,^^f^	0	1 (3.33%)	9 (30.00%)	13 (43.33%)	7 (23.33%)	30
	15**–**18^c^^,^^e^^,^^f^	0	0	0	1 (2.50%)	39 (97.50%)	40
Female	6**–**8^a^^,^^b^^,^^c^	22 (73.33%)	8 (26.67%)	0	0	0	30
	9**–**11^a^^,^^d^^,^^e^	1 (3.33%)	15 (50.00%)	13 (43.33%)	1 (3.33%)	0	30
	12**–**14^b^^,^^d^^,^^f^	0	0	3 (10.00%)	17 (56.67%)	10 (33.33%)	30
	15**–**18^c^^,^^e^^,^^f^	0	0	0	1 (2.50%)	39 (97.50%)	40

MPSS, maturation stage of midpalatal suture.

a Indicates a significant difference of the MPSS distribution pattern between 6 and 8 group and 9–11 group (*p* < 0.05).

b Indicates a significant difference of the MPSS distribution pattern between 6 and 8 group and 12–14 group (*p* < 0.05).

c Indicates a significant difference of the MPSS distribution pattern between 6 and 8 group and 15–18 group (*p* < 0.05).

d Indicates a significant difference of the MPSS distribution pattern between 9 and 11 group and 12–14 group (*p* < 0.05).

e Indicates a significant difference of the MPSS distribution pattern between 9 and 11 group and 15–18 group (*p* < 0.05).

f Indicates a significant difference of the MPSS distribution pattern between 12 and 14 group and 15–18 group (*p* < 0.05).

### 3.2 Zygomaticomaxillary suture maturation stage in adolescent subjects

Similar to MPSS, ZMSS also presents a progressively developing tendency from stage A to stage E in male and female adolescents aged 6–18 (Figure 2B). In males and females aged 6–8, the majority of individuals were classified as stage A, with a few transitioning to stage B. During the age of 9–11, males showed stages A, B, and C of ZMSS; while females exhibited stages A, B, C, and D with higher proportions observed for stages B and C.; The most significant variation in ZMSS was observed in males aged 12–14 where distribution included stage A, B, C, D, and E with higher proportions seen for stage C and D. Females displayed distributions primarily consisting of stages B, C, D, and E with greater prevalence in stages D and E. Both genders aged 15–18 years old are at stage E. And there is a statistically significant difference in the distribution of ZMSS between different age groups (Table 2).

**Table 2:** The distribution of ZMSS at different ages in all subjects.

Gender	Age	ZMSS					Total
		A	B	C	D	E	
Male	6**–**8^a^^,^^b^^,^^c^	25 (83.33%)	5 (16.67%)	0	0	0	30
	9**–**11^a^^,^^d^^,^^e^	4 (13.33%)	19 (63.33%)	7 (23.33%)	0	0	30
	12**–**14^b^^,^^d^^,^^f^	1 (3.33%)	1 (3.33%)	12 (40.00%)	12 (40.00%)	4 (13.33%)	30
	15**–**18^c^^,^^e^^,^^f^	0	0	1 (2.50%)	6 (15.00%)	33 (82.50%)	40
Female	6**–**8^a^^,^^b^^,^^c^	25 (83.33%)	5 (16.67%)	0	0	0	30
	9**–**11^a^^,^^d^^,^^e^	2 (6.67%)	14 (46.67%)	13 (43.33%)	1 (3.33%)	0	30
	12**–**14^b^^,^^d^^,^^f^	0	1 (3.33%)	2 (6.67%)	15 (50.00%)	12 (40.00%)	30
	15**–**18^c^^,^^e^^,^^f^	0	0	0	3 (7.50%)	37 (92.50%)	40

ZMSS, maturation stage of zygomaticomaxillary suture.

a Indicates a significant difference of the MPSS distribution pattern between 6 and 8 group and 9–11 group (*p* < 0.05).

b Indicates a significant difference of the MPSS distribution pattern between 6 and 8 group and 12–14 group (*p* < 0.05).

c Indicates a significant difference of the MPSS distribution pattern between 6 and 8 group and 15–18 group (*p* < 0.05).

d Indicates a significant difference of the MPSS distribution pattern between 9 and 11 group and 12–14 group (*p* < 0.05).

e Indicates a significant difference of the MPSS distribution pattern between 9 and 11 group and 15–18 group (*p* < 0.05).

f Indicates a significant difference of the MPSS distribution pattern between 12 and 14 group and 15–18 group (*p* < 0.05).

### 3.3 Correlations among ZMSS, MPSS and CVMS

**Figure 3:**
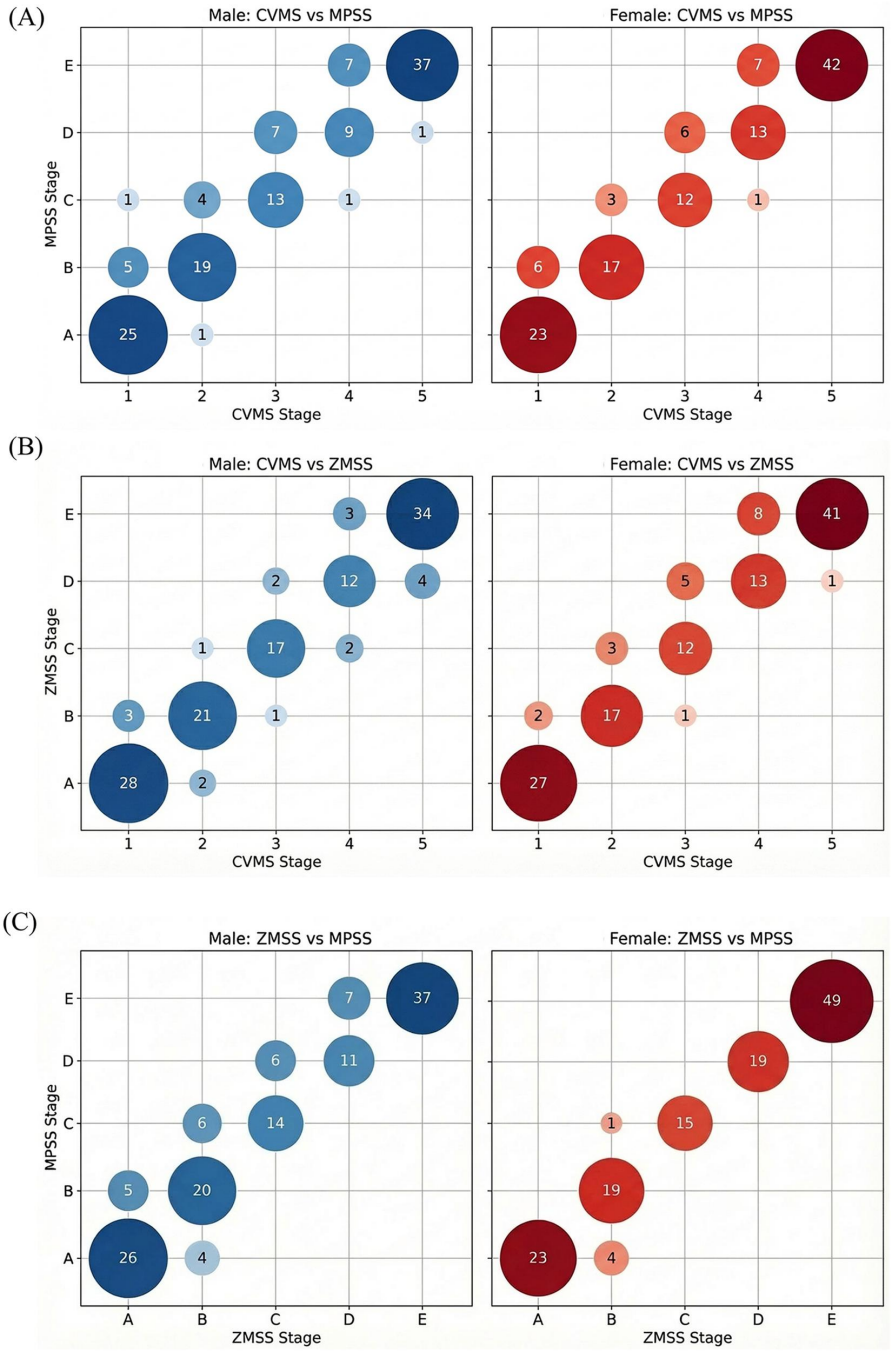
Sex-stratified bubble plots depicting the joint distributions among CVMS, MPSS, and ZMSS stages. Bubble area is proportional to the number of subjects with the corresponding pair of stages, and bubble color intensity increases with frequency. **(A)** Maturation stage of cervical vertebrae (CVMS) vs. maturation stage of midpalatal suture (MPSS). **(B)** CVMS vs. maturation stage of zygomaticomaxillary suture (ZMSS). **(C)** ZMSS vs. MPSS. The underlying sex-specific contingency data are provided in Tables 3–5.

Correlation analysis among CVMS, MPSS and ZMSS revealed statistically significant positive correlations between each pair of indicators in both genders, confirming a synchronous developmental pattern (Figure 3). As shown in Table 3, CVMS and MPSS were strongly correlated. The distributions demonstrated clear stage-wise alignment, with each successive CVMS stage predominantly corresponding to the next MPSS stage (e.g., CVMS1 with MPSS A, CVMS5 with MPSS E). Similarly, CVMS and ZMSS exhibited a high correlation, with a consistent progressive association across stages (Table 4). Most notably, MPSS and ZMSS showed an exceptionally strong correlation. The contingency table (Table 5) revealed a near-perfect stage-to-stage correspondence, particularly in female subjects where multiple stages (D, E) showed complete alignment.

**Table 3:** Correlation between CVMS and MPSS.

Gender	CVMS	MPSS					Total	Correlation coefficient
		A	B	C	D	E		
Male	1	25	5	1	–	–	31	0.956*
	2	1	19	4	–	–	24	
	3	–	–	13	7	–	20	
	4	–	–	1	9	7	17	
	5	–	–	–	1	37	38	
	Total	26	24	19	17	44	130	
Female	1	23	6	–	–	–	29	0.965*
	2	–	17	3	–	–	20	
	3	–	–	12	6	–	18	
	4	–	–	1	13	7	21	
	5	–	–	–	–	42	42	
	Total	23	23	16	19	49	130	

CVMS, maturation stage of cervical vertebrae; MPSS, maturation stage of midpalatal suture.

* Indicates *P* < 0.001.

**Table 4:** Correlation between CVMS and ZMSS.

Gender	CVMS	ZMSS					Total	Correlation coefficient
		A	B	C	D	E		
Male	1	28	3	-	–	–	31	0.967*
	2	2	21	1	–	–	24	
	3	–	1	17	2	–	20	
	4	–	–	2	12	3	17	
	5	–	–	–	4	34	38	
	Total	30	25	20	18	37	130	
Female	1	27	2	–	–	–	29	0.965*
	2	–	17	3	–	–	20	
	3	–	1	12	5	–	18	
	4	–	–	–	13	8	21	
	5	–	–	–	1	41	42	
	Total	27	20	15	19	49	130	

CVMS, maturation stage of cervical vertebrae; ZMSS, maturation stage of zygomaticomaxillary suture.

* Indicates *P* < 0.001.

**Table 5:** Correlation between MPSS and ZMSS.

Gender	ZMSS	MPSS					Total	Correlation coefficient
		A	B	C	D	E		
Male	A	26	–	–	–	–	26	0.970*
	B	4	20	–	–	–	24	
	C	–	5	14	–	–	19	
	D	–	–	6	11	–	17	
	E	–	–	–	7	37	44	
	Total	30	25	20	18	37	130	
Female	A	23	-	-	–	–	23	0.993*
	B	4	19	-	–	–	23	
	C	–	1	15	–	–	16	
	D	–	–	–	19	–	19	
	E	–	–	–	–	49	49	
	Total	27	20	15	19	49	130	

MPSS, maturation stage of midpalatal suture; ZMSS, maturation stage of zygomaticomaxillary suture.

* Indicates *P* < 0.001.

### 3.4 Diagnostic performance of CVMS and MPSS

The positive LHRs for the identification of suture maturation stages are summarized in Tables 6–8. Notably, in both genders, CVMS1, CVMS2, CVMS3, and CVMS5 showed LHR values >10 for corresponding MPSS stages (A, B, C, and E, respectively), indicating a strong diagnostic association. In males, all CVMS stages yielded LHRs >10 for diagnosing ZMSS, whereas in females, CVMS4 showed moderate utility for detecting ZMSD. Importantly, MPSS demonstrated consistently high LHRs (≥10) for predicting ZMSS across all stages, reinforcing its role as a reliable surrogate indicator when ZMSS assessment is challenging.

**Table 6:** Positive likelihood ratio of CVMS for identification of MPSS.

Gender	CVMS	MPSS				
		A	B	C	D	E
Male	1	16.67	0.8494	0.1947	–	–
	2	0.1739	16.78	1.168	–	–
	3	–	–	10.85	3.579	–
	4	–	–	0.3651	7.478	1.368
	5	–	–	–	0.1797	72.32
Female	1	17.83	1.214	–	–	–
	2	–	26.36	1.257	–	–
	3	–	–	15.50	2.921	–
	4	–	–	0.3563	9.493	0.8265
	5	–	–	–	0.7320	+∞

CVMS, maturation stage of cervical vertebrae; MPSS, maturation stage of midpalatal suture.

**Table 7:** Positive likelihood ratio of CVMS for identification of ZMSS.

Gender	CVMS	ZMSS				
		A	B	C	D	E
Male	1	31.11	0.450	–	–	–
	2	0.3030	29.4	0.25	–	–
	3	–	0.2211	31.17	0.7037	–
	4	–	–	0.7333	14.933	0.5386
	5	–	–	–	0.7320	21.365
Female	1	51.5	0.4074	–	–	–
	2	–	31.17	1.353	–	–
	3	–	0.3235	15.33	2.247	–
	4	–	–	–	9.493	1.017
	5	–	–	–	0.1424	67.78

CVMS, maturation stage of cervical vertebrae; ZMSS, maturation stage of zygomaticomaxillary suture.

**Table 8:** Positive likelihood ratio of MPSS for identification of ZMSS.

Gender	MPSS	ZMSS				
		A	B	C	D	E
Male	A	26	0.6795	–	–	–
	B	–	17.67	1.461	–	–
	C	–	–	13.63	2.849	–
	D	–	–	–	10.45	1.244
	E	–	–	–	–	+∞
Female	A	26.75	0.8091	–	–	–
	B	–	88.39	0.3750	–	–
	C	–	–	+∞	–	–
	D	–	–	–	+∞	–
	E	–	–	–	–	+∞

MPSS, maturation stage of midpalatal suture; ZMSS, maturation stage of zygomaticomaxillary suture.

## 4 Discussion

The reliability of CVMS in evaluating the pubertal peak and skeletal growth had been demonstrated by extensive researches (13, 14). In orthodontics, CVMS are commonly adopted in predicting the maturation stage of circummaxillary sutures, but several researchers suggested that CVMS is more consistent with the growth of mandible rather than maxilla (16). Beit et al*.* concluded that cervical spine evaluation offers no advantage over chronologic age in either assessing skeletal age or predicting the pubertal growth spurt (18). Therefore, exploring new approaches to assess the growth stage of maxilla is essential for evaluating the optimum timing for maxillary expansion therapy.

Previous researches showed that success of MP/TPE is strongly related to the maturation of circum-maxillary sutures. Among these sutures, the orientations of ZMSS and MPSS closely align with the directions of the applied force system used in MP/TPE procedures. Recent studies suggested that great variability exists among individuals due to factors such as the population's ethnicity, nutritional conditions, and surrounding anatomic structures (20, 21). Therefore, assessing the suture maturation of MPS and ZMS can be promising in evaluating skeletal age and predicting individual responses to maxillary protraction.

In this research, the correlation analysis among ZMSS, MPSS and CVMS showed that significant (*P* < 0.05) positive correlations exist in any two kinds of maturation stages regardless of gender, indicating a shared growth pattern similar to the findings reported by Angelieri et al. (19, 24). While CVMS can generally serve as a reliable predictor for identifying MPSS and ZMSS, careful consideration is still necessary when deciding whether to proceed with MP/TPE treatment on patient at CVMS3. Notably, our observations indicate that minors at CVMS3 stage exhibited MPSD transformation in 10% of male subjects and 27.7% of female subjects, suggesting sutural fusion had occurred in certain regions. In such cases, where sutural fusion has begun, the orthopedic forces exerted by MP/TPE may predominantly induce dental changes rather than desired maxillary skeletal changes. This biomechanical challenge is consistent with reports from other maxillary expansion modalities, where high skeletal resistance has been associated with an increased risk of adverse dentoalveolar effects, such as alveolar bone loss and gingival recession (9, 27, 28).

LHR is a parameter for the diagnostic value of a new indicator. An LHR >10 indicates a conclusive increase (over 45%) in the post-test probability of a condition, which supports the reliability of the diagnostic indicator (29). In terms of diagnosing MPSS in male subjects, we observed an LHR>10 between CVMS1 and MPSA, CVMS2 and MPSB, CVMS3 and MPSC, as well as CVMS5 and MPSE. Angelieri et al.'s study reported a positive LHR of 11.3 for CVMS3 to MPSC, which aligns with our findings in male subjects but is lower than that observed in female subjects. This discrepancy may originate from the differences in the ethnicity of the chosen sample (23). The LHR between CVMS4 and MPSE is 7.478, indicating moderate confidence level; thus suggesting greater variability in MPSS among individuals with CVMS4 (Table 6). Similar results were also presented in female subjects, but the LHR for diagnosing MPSE using CVMS5 is +∞, implying that all cases of MPSS in females at CVMS5 are classified as MPSE (Table 8). Our results confirm the findings of Angelieri et al. regarding the strong correlation between MPS and ZMS maturation. However, the novelty of our work lies in establishing these correlations within a Chinese adolescent population, for whom detailed data was previously lacking. The positive likelihood ratios we observed for diagnosing ZMSS based on MPSS were consistently high, even stronger in females, which provides a robust, population-validated tool for clinicians. This is significant given the recognized ethnic variability in craniofacial growth patterns.

The diagnosis of ZMSS is more intricate than that of MPSS because it requires the evaluator to rotate the patient's head in multiple planes to adequately visualize both the inferior and posterior portions of the suture. The diagnostic performance of MPSS in diagnosing ZMSS consistently exhibited a positive LHR of 10 or higher at each stage, indicating its reliability as a diagnostic aid in future clinical perspective. We noticed that the correlation between CVM and maturation stages of ZMS was demonstrated to be high, which agrees with previous studies on MPS (23, 30). Female subjects displayed an infinite LHR between MPSC and ZMSC, MPSD and ZMSD, as well as MPSE and ZMSE, highlighting a more significant coordinated developmental pattern between MPS and ZMS in females compared to their male counterparts. In addition, since MPSC is present in CVMS4 in both genders, orthodontists shouldn't rule out the possibility of MP/TPE therapy in CVMS4 patients and CBCT examination is recommended the further confirm MPSS (Table 3). By considering both CVMS and MPSS, orthodontists can minimize side effects or avoid failure of the treatment for younger patients with delayed MPSS. Notably, Angelieri et al. suggested that ZMS on both sides should be examined since different stages may be observed on each side for certain patients; thus, the treatment planning should be based on the more matured side of ZMS (19). To sum up, given the high correlation between MPSS and ZMSS, MPSS assessment can provide valuable inferential information about ZMSS status. However, when CBCT is available, direct evaluation of ZMSS remains the most accurate method. Clinically, integrating both MPSS and ZMSS assessments can enhance diagnostic precision for timing maxillary protraction and expansion therapy. Based on our findings, clinical recommendations can be stratified according to available imaging resources: In settings where CBCT is accessible, direct evaluation of ZMSS and MPSS should be prioritized for assessing circum-maxillary suture maturation. These direct measurements allow for precise staging and individualized timing of maxillary protraction and expansion, particularly in patients with suspected advanced suture fusion. In clinics limited to lateral cephalograms, CVMS offers a practical and correlated indicator of suture maturation. Although indirect, CVMS stages, especially CVMS1–CVMS3, show strong positive likelihood ratios for corresponding MPSS/ZMSS stages and can guide preliminary timing decisions, with consideration for CBCT referral if suture fusion is suspected. It is noteworthy that a potential limitation of this study is its cross-sectional design, which establishes correlation but not causation. While we have demonstrated a strong association between suture maturation stages and growth indicators, the direct correlation between specific MPS/ZMS stages and the actual orthopedic outcomes of MP/TPE was not assessed, and we did not model potential confounders such as skeletal phenotype and individual growth status. A future longitudinal study investigating the treatment efficacy of MP/TPE relative to the pre-treatment MPSS and MPSS in Chinese adolescents, while prospectively measuring and statistically controlling for sex, age, skeletal phenotype, and growth status, would be invaluable to further enhance the clinical significance of these diagnostic classifications.

In clinical practice, the classification method of MPSS and ZMSS proposed by Angelieri seems simple for orthodontists; however, the subjective nature and varying observation methods may lead to inconsistent judgments on suture maturation stage (24). Barbose et al. indicated that despite individual assessment of midpalatal suture maturation revealed potential reliability and reproducibility, the agreement rate was insufficient for routine clinical application (31). Recently, great advancements have been achieved in the field of deep learning systems-based diagnosis in oral and craniofacial imaging. Convolutional neural networks (CNN) have shown ideal accuracy in identifying anatomic structures through feature extraction from extensive data sets. Kim et al. proposed a three-step deep-learning model involving region of interest detection, cervical segmentation and CVMS classification, which achieved an accuracy of 62.5% in estimating CVMS using lateral cephalograms (32). Zhu et al*.* applied CNN models in assessing the maturation stage of the midpalatal suture by training the model with 785 CBCT scans with 2,371 images. Among all the models, Resnet50 demonstrated an outstanding accuracy of 99.74% in identifying the transverse plane that contained the complete midpalatal suture, which exceeded the average accuracy of three experienced orthodontists (33). We believe that a similar deep-learning model could also be adopted to evaluate skeletal maturity based on ZMSS. Furthermore, a more comprehensive and individualized assessment might be achieved by simultaneously analyzing the CVMS, MPSS, and ZMSS.

## 5 Conclusions

This study demonstrates that CBCT-based assessment of ZMS maturation serves as a reliable clinical indicator for evaluating skeletal maturity. Significant correlations were confirmed among ZMSS, MPSS, and CVMS, supporting their synergistic use in diagnostics. The high positive LHRs support the integrated use of CBCT-based suture assessment and CVMS for evaluating skeletal maturity. Orthodontists should consider both MPSS and ZMSS to optimize the timing of maxillary protraction and expansion therapy for adolescents with transverse maxillary deficiency.

## Data Availability

The original contributions presented in the study are included in the article/Supplementary Material, further inquiries can be directed to the corresponding authors.
